# Pathophysiological Mechanisms to Review Association of Atrial Fibrillation in Heart Failure With Obstructive Sleep Apnea

**DOI:** 10.7759/cureus.16086

**Published:** 2021-07-01

**Authors:** Pushyami Satya Bandi, Preetish Kumar Panigrahy, Sreehita Hajeebu, Ngonack J Ngembus, Stacey E Heindl

**Affiliations:** 1 Medicine, California Institute of Behavioral Neurosciences & Psychology, Fairfield, USA; 2 Pharmacology, California Institute of Behavioral Neurosciences & Psychology, Fairfield, USA; 3 Medicine, California Institute of Behavioural Neurosciences & Psychology, Fairfield, USA; 4 Medicine, Avalon University School of Medicine, Willemstad, CUW

**Keywords:** sleep apnea, sleep dyspnea, atrial fibrillation, a-fib, abnormal rhythm, arrhythmia, cardiac diseases, heart failure, heart complications, obstructive sleep apnea

## Abstract

Sleep-disordered breathing (SDB) comprising obstructive sleep apnea (OSA) is found in more than half of patients with heart failure (HF) and may have negative impacts on cardiovascular function. Increased atherosclerotic cardiovascular disease and the development of coronary events and congestive heart failure are associated with OSA. It is associated with a substandard quality of life, increased hospitalizations, and a poor prognosis. Despite its association with severe cardiovascular morbidity and mortality, the condition is frequently underdiagnosed. The substantial clinical evidence has established OSA as an independent risk factor for bradyarrhythmias and tachyarrhythmias in the last decade. The mechanisms which lead to such arrhythmias are uncertain. In short, OSA patients have a significantly elevated risk of HF and atrial fibrillation (AF). The direct correlation between HF, SDB, and cardiac arrhythmias has been poorly understood. The purpose of this study is to get a better understanding of the relation between AF, OSA, and HF by focusing on the pathophysiological mechanisms underlying these conditions. Therefore, we searched for articles to support our association in PubMed and Google Scholar databases.

## Introduction and background

There is a diverse yet poorly defined relationship between heart failure (HF), sleep-disordered breathing (SDB), and cardiac arrhythmias [1].

HF is a significant and rapidly increasing public health problem [2,3]. In developed countries, HF affects about 2% of the entire adult population, a rate that rises to 10% in those over 70 years of age [4,5]. It is a significant cause of mortality and morbidity and is associated with increasingly severe symptoms such as shortness of breath, tiredness, edema as well as chronic disease, and reduced quality of life [2,6,7].

In patients with HF, SDB is increasingly recognized as a predictor of poor prognosis and a disease process that may speed up the decline of heart function [4]. More than nearly half of HF patients (with either retained or decreased ejection fraction) have SDB, which is about ten times the population average [4,8-10]. In the absence of indications, such as prolonged daytime sleepiness, screening for SDB is not usually done in HF patients [1]. Therefore, in patients with HF, sleep apnea remains a widely underdiagnosed disorder [1].

Central sleep apnea (CSA), obstructive sleep apnea (OSA), or a combination of the two are examples of SDB [4]. According to mounting proof, OSA is implicated in both the onset and further worsening of atrial fibrillation (AF) [11].

Population-based studies identified numerous factors that change the atrial substrate (structural changes of atrium) and increase AF vulnerability [12]. Modifiable risk factors include smoking, sedentary lifestyle, obesity, OSA, and elevated blood pressure, and each factor is said to cause structural and electrical atrial remodeling [12]. Among them, a significant risk factor for the development of AF is OSA [12]. Both HF and myocardial infarction also increase the risk of AF and vice versa, generating a mortality-increasing process [12].

## Review

Despite existing research on the mechanisms of HF, OSA, and AF, evidence on the relationship between AF and OSA in HF patients is lacking. The pathophysiological pathways that predispose to the development of OSA and AF and how they are affected in HF patients will be reviewed and discussed in this article. We used PubMed and Google Scholar to find 46 articles relevant to our subject. We included ten papers after filtering papers written entirely in English and published during the last ten years.

Classification of sleep-disordered breathing and definitions

OSA, CSA, or a combination of both are part of SDB [4]. OSA characterizes by partial or complete airway obstruction episodes lasting > 10s during sleep [13]. CSA causes intermittent cessation of inspiratory drive due to a drop below the apnea threshold in PaCO2 (partial pressure of carbon dioxide) [14,15].

Currently, apnea is characterized by reduced airflow by at least 90% of the baseline pre-event for at least 10 seconds [4,16]. Hypopnea is characterized by a reduction in the airflow by at least 30% from the baseline for at least 10s associated with a decrease in arterial oxygen saturation of at least 3% or sleep arousal [4,16]. The 'apnea-hypopnea index' (AHI) is the mean number of apnoeic and hypopnoeic occurrences per hour of sleep [4]. It is 'natural' for up to five events per hour, 5-15/h as 'mild,' 15-30/h as 'moderate,' and > 30/h as 'extreme' SDB [4]. Patients with > 50 percent of events as obstructive are classified as 'predominantly' OSA, and a patient labeled as' predominantly 'CSA if > 50 percent of events are central [4].

Interaction between obstructive sleep apnea and heart failure

**Figure 1:**
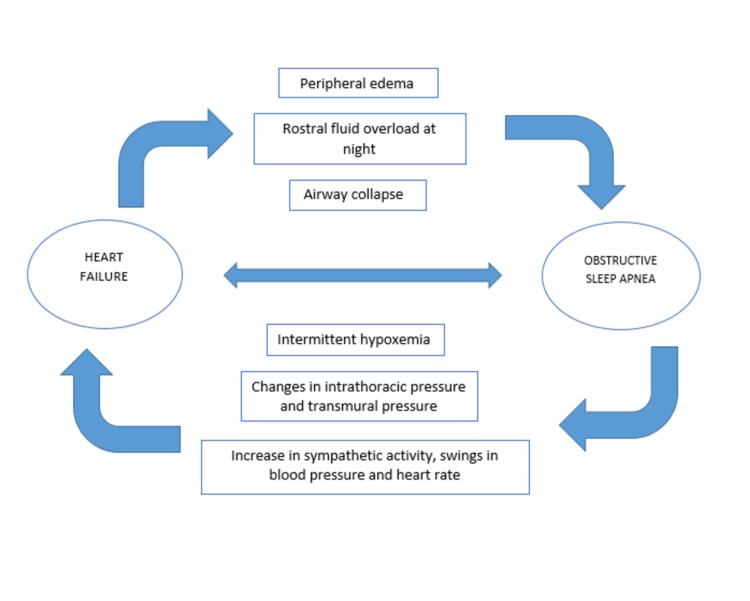
Interaction of heart failure and obstructive sleep apnea.

OSA can, in many ways, accelerate the development of HF, as shown in Figure 1.

During inspiration, in cases of OSA, the negative intrathoracic pressure created by the respiratory muscles attempting to inspire against a closed airway increases venous return to the right heart, increases preload, and causes the septum to move to the left, which may compromise the role of the left ventricle (LV) [4]. During episodes of negative intrathoracic pressure, which raises the afterload, enhanced preload further compromises the capacity of the failing LV to cope by increasing transmural pressure [4]. Apnea and hypopnea stimulate the sympathetic nervous system [4]. In those with OSA and HF, serum catecholamines and muscle sympathetic nerve activity are higher than matched controls with HF only [4,17,18]. Patients with OSA also experience variations in blood pressure and heart rate, which affect shear stress on the vascular endothelium and can contribute to endothelial dysfunction in conjunction with persistent hypoxemia [4,19].

In men without HF, we found that the amount of fluid displaced overnight from the legs correlated strongly with a rise in the circumference of the neck overnight and the frequency of hypopnea and obstructive apnea per hour of sleep (i.e., AHI) [14,20]. Hence fluid displaced from the legs into the neck increased the tendency for pharyngeal obstruction [14]. Therefore, it is conceivable that overnight rostral fluid displacement into the neck in patients with HF, a disorder characterized by dependent fluid retention in the legs, might also lead to OSA pathogenesis [14].

OSA is related to a rise in sympathetic activity, and this further contributes to extra peripheral vasoconstriction, tachycardia, and salt and water retention stimulation of the renin-angiotensin-aldosterone system (RAAS) [4]. The damping of this response is considered essential for improving the long-term prognosis in the neurohormonal model of HF [4]. It perhaps comes as no surprise, given the pathophysiological effects of SDB, that SDB is associated with poor outcomes in people with HF and the general population [4]. For almost nine years, the Sleep Heart Health study tracked 4422 patients free of heart disease at baseline [4,21]. During follow-up, those with severe OSA had more than double the all-cause mortality [4,21].

Therefore, in addition to the effects of neurohormonal modulators such as beta-blockers, angiotensin-converting enzyme (ACE) inhibitors, and aldosterone antagonists, SDB could be a potential therapeutic target for HF [4].

Association of atrial fibrillation with obstructive sleep apnea

Given the high incidence of sleep apnea in HF patients, it is appropriate to inquire about any irregular breathing patterns during sleep from the patient's partner [11]. When a new arrhythmia, such as AF, is discovered in a patient with HF, a sleep apnea test is recommended [11]. OSA may be assumed based on medical history (e.g., snoring, witnessed apneas, waking up with a choking feeling, and extreme daytime sleepiness) and physical evaluation (e.g., increased neck circumference, short neck) [11,22]. Overnight polysomnography, which monitors airflow, respiratory muscle function, electroencephalography, electrocardiogram, and blood pressure, is the gold standard for diagnosing sleep apnea [11].

AF is significantly more common in patients with OSA than in those without OSA, according to several findings [11,23-26]. Arrhythmias become more frequent as OSA becomes more profound [11]. A summary of studies indicating an increased risk of AF in OSA patients can be found in Table 1.

**Table 1:** Summary of studies indicating an increased risk of AF in OSA patients. PSG-polysomnography, OR-odds ratio, AF-atrial fibrillation, OSA-obstructive sleep apnea, HR-hazard ratio Modified from Latina JM et al. [26]

Name of the Study	Number of participants	The method used to diagnose OSA	Results
Guilleminault et al. [27]	400	PSG	The study found cardiac arrhythmias in 48 percent of OSA patients.
Mehra et al. [28]	566	PSG	The adjusted OR for the risk of AF in OSA patients was 4.02 (1.03–15.74).
Gami et al. [29]	3542	PSG	Incidence of AF in OSA patients under the age of 65, HR 3.29 (1.35-8.04)
Tanigawa et al. [30]	1763	Pulse oximeter during sleep	The adjusted odds ratio (OR) for AF in patients with severe OSA is 5.66 (1.75–18.34).
Monahan et al. [31]	2816	PSG	Compared to normal breathing, the risk of AF after a respiratory disturbance is 17.9 (2.2–144.2).

Using 24-hour Holter monitoring, one of the first studies looked at the frequency of cardiac arrhythmias and conduction disturbance in 400 patients with OSA [11,27]. In this group of patients, Guilleminault et al. discovered a slight non-statistically significant rise in the incidence of paroxysmal AF [11,27]. However, most research found that patients with OSA had a far higher prevalence of AF [11,27]. Mehra et al. found that the risk for AF in severe OSA is about four times as high as that of those without OSA [11,28]. Gami et al. studied 3,542 Olmsted County adults with OSA diagnosed by polysomnogram in a retrospective cohort sample. During a 4.7-year follow-up, electrocardiography confirmed the existence of new-onset AF [11,29]. 

Mechanism of atrial fibrillation in obstructive sleep apnea

**Figure 2:**
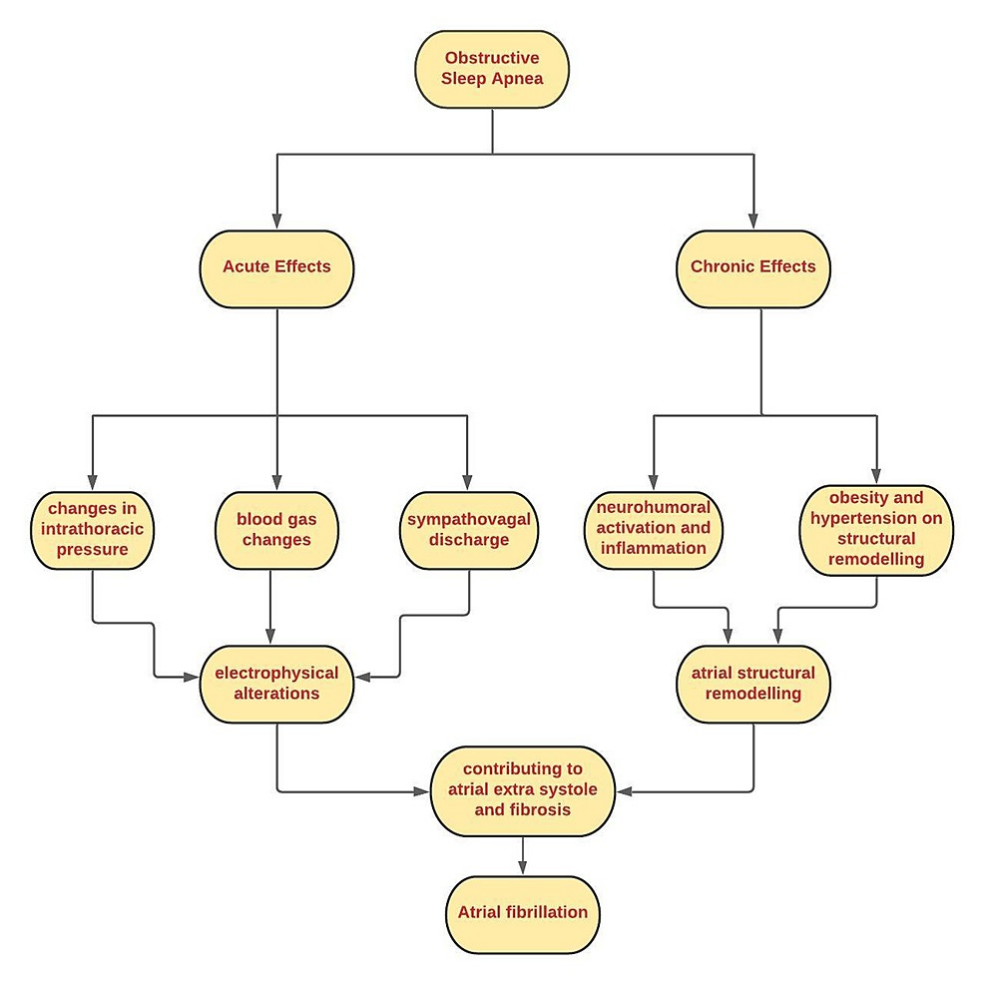
The acute and long-term effects of obstructive sleep apnea.

A variety of pathophysiological mechanisms may cause both OSA and AF; thus, the presence of one may promote the existence of the other [11]. The mechanisms can be acute and chronic changes concerning OSA [31]. Figure 2 summarizes possible mechanisms involved in the onset and persistence of AF in OSA patients.

Acute factors of OSA effecting onset of atrial fibrillation

Acute factors directly associated with obstructive respiratory events can contribute significantly to the incidence of AF in OSA, such as changes in intrathoracic pressure, changes in blood gases, and sympathovagal imbalance [31].

*Changes in intrathoracic pressure*: Obstructed inspirations cause significant intrathoracic pressure variations, resulting in changes in the transmural pressure of the heart, resulting in atrial stretch [31]. Arrhythmogenic atrial electrophysiological alterations can result from acute atrial dilation caused by thoracic pressure fluctuations [31]. Orban and colleagues used the Mueller maneuver to replicate OSA in 24 healthy young adults [11,32]. The Mueller maneuver entails forcing air into the lungs through a closed mouth and nose to create a significant negative pressure in the chest [11]. They discovered that the left atrial volume decreased significantly during the procedure, and the LV end-systolic dimension increased, indicating that the LV ejection fraction decreased [11]. Following the maneuver, blood flow, stroke volume, ejection fraction, and cardiac production increased above usual [11]. Repetitive swings in afterload burden and chamber volumes, they hypothesized, may also have outcomes for AF and coronary heart failure development within the future [11].

*Blood gas changes*: OSA causes frequent episodes of hypoxia and hypercapnia, which activate the chemoreflex and increase sympathetic nerve activity, causing tachycardia and high blood pressure, particularly at the end of apneic attacks [11,27,33]. Myocardial oxygen demand rises due to tachycardia and hypertension, while myocardial oxygen supply falls due to hypoxia causing recurrent myocardial ischemia while sleeping, which induces atrial and ventricular fibrosis, arrhythmias in the atrium and ventricles, as well as sudden cardiac death [11,33,34].

*Sympatho-vagal imbalance*: Direct muscle sympathetic nerve activity recordings showed increased sympathetic activation in OSA patients during apnea episodes [31,35]. Linz et al. found that in a pig model of OSA, the negative intratracheal pressure produced by forced inspiration triggered the parasympathetic nervous system that is known to stimulate afferent vagal fibers in the thorax [11,36,37].

Long-term effects of OSA on atrial structural remodeling

Major atrial remodeling marked by 'atrial enlargement, local conduction disruptions and longer sinus node recovery,' atrial electromechanical delay, and left atrial dysfunction, is correlated with long-term OSA in patients [31,34,38]. Several mechanisms, including systemic inflammation, neurohumoral activation, and persistent atrial dilation by repeated changes in intrathoracic pressure and multiple comorbidities such as obesity and hypertension, have been considered to cause OSA-related myocardial damage [31].

Neurohumoral activation and inflammation: OSA is related to elevations of circulating inflammation markers, and in patients who may undergo atrial structural and electrical remodeling, neurohumoral activation, namely the circulating RAAS combined with increased oxidative stress, has been demonstrated [31,39-42].

Impact of obesity and hypertension on structural remodeling: In the emergence of hypertension and the evolution of drug-resistant hypertension, OSA is considered an etiological factor [31,43,44]. Chronically high blood pressure was associated with severe atrial electrical and structural remodeling in a sheep model [31,45]. Characterized by local conduction disorders, atrial wavelength shortening, and increased AF occurrence, atrial myocyte hypertrophy and myolysis, increased atrial collagen, and apoptosis [31,45].

Obstructive sleep apnea and atrial fibrillation in heart failure

HF causes pulmonary congestion by increasing LV wall tension and LV filling pressures, stimulating lung irritant vagal receptors [1]. The Cheyne-Stokes respiration pattern is maintained in HF patients with alternating hyperventilation and apnea by a complex interaction of pulmonary congestion caused by central fluid accumulation, triggered by increased respiratory chemoreceptor drive, apnea-induced hypoxemia, increased venous return in the supine position, and arousals that cause oscillations in the arterial carbon dioxide level beyond the normal range [1].

**Figure 3:**
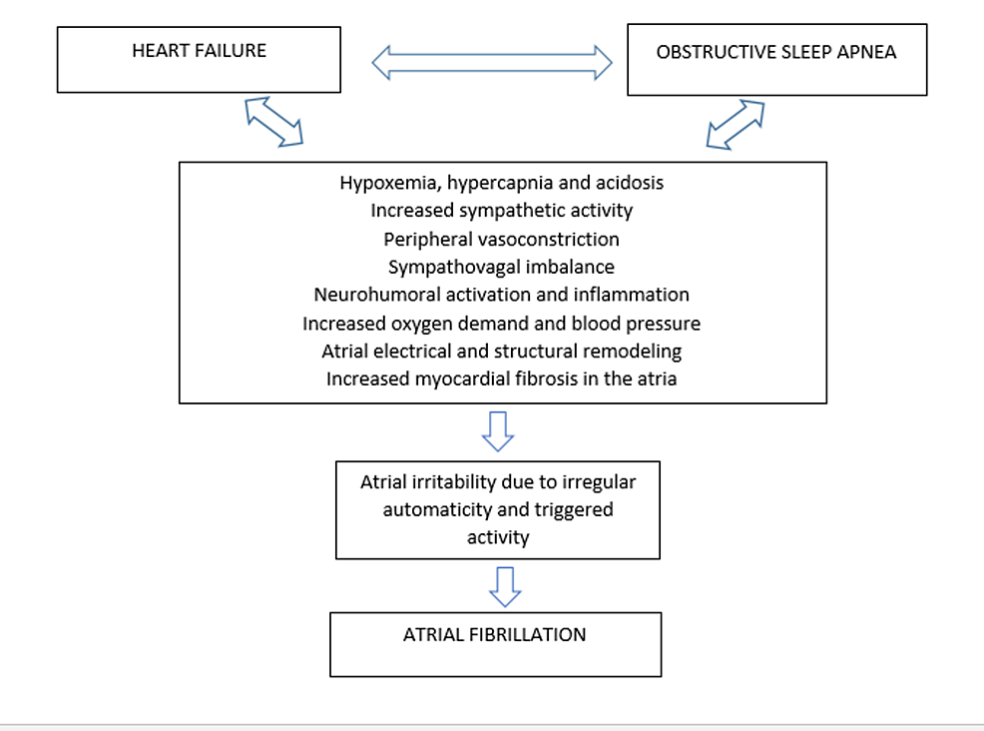
Pathophysiological cycle showing the association of heart failure and obstructive sleep apnea with atrial fibrillation.

In HF patients, OSA enhances sympathetic nervous system function, followed by several neurohumoral and hemodynamic responses, all of which contribute to the stress on the failing heart [1]. Due to irregular automaticity and activated activity, apneas cause intermittent elevations in sympathetic activity and parasympathetic withdrawal, favoring atrial and ventricular irritability [1,46]. Increased sympathetic activity may cause tachycardia, peripheral vasoconstriction, and renin-angiotensin system activation due to increased oxygen demand, blood volume, myocardial oxygen demand, and blood pressure [1,47]. This sequence of events could lead to a pathophysiological vicious circle, as in Figure 3 [1,47]. Lower intrathoracic pressures accompany increased inspiratory efforts between apnea episodes, contributing to increased left ventricular pressure, wall tension, and afterload [1]. Furthermore, SDB has been related to increased amounts of various markers of inflammation, oxidative stress, endothelial dysfunction, platelet dysfunction, and myocardial ischemia in HF patients, which may induce myocyte necrosis and apoptosis, and thus increase the amount of myocardial fibrosis in both the atria and the ventricles [1]. The most common mechanism of sustained AF is diffuse myocardial fibrosis in both atria, facilitating reentry by multiple wavelets [1].

## Conclusions

This paper aims to look into the pathophysiological mechanisms underlying OSA and AF in HF patients. We were able to demonstrate that OSA is associated with HF and contributes to AF through various means, including neurohumoral and hemodynamic changes in sympathetic, atrial structure, and electrical activity, as explained in the paper. Accordingly, cardiologists should have a high suspicion index for SDB in patients with HF and collaborate closely with sleep doctors to enhance patient care. As a result, it will be crucial for medical professionals to understand the mechanisms related to sleep apnea and cardiac arrhythmias in HF and to include them in diagnosis, since they are often underdiagnosed when considered independently. In this way, we can avoid complicated AF treatments and concentrate on sleep treatment to improve conditions related to sleep apnea. Early detection of risk factors can also help patients reduce their risk for AF and worsening of HF.
